# A Novel Method in Surface Water Quality Assessment Based on Improved Variable Fuzzy Set Pair Analysis

**DOI:** 10.3390/ijerph16224314

**Published:** 2019-11-06

**Authors:** Yucheng Liu, Chuansheng Wang, Yutong Chun, Luxin Yang, Wei Chen, Jack Ding

**Affiliations:** 1 School of Information, Capital University of Economics and Business, Beijing 100070, China; 12018207067@cueb.edu.cn (C.W.);; 2 State Key Laboratory, Nanjing University of Finance and Economics, Nanjing 210023, China; 3 Institute of International Economy, University of International Business and Economics, Beijing 100029, China; 4 School of Arts and Science, University of Toronto, Toronto, ON M5S2E8, Canada

**Keywords:** surface water pollution, water quality assessment, IVFSPA method, game comprehensive weighting method, improved Nemerow method, double judgment mode

## Abstract

In the case of surface water pollution, it is important and necessary to accurately assess the level of contaminated water and ensure the safety of drinking water for people in disaster areas during floods. However, for the assessment of the strict requirements of drinking water, traditional assessment methods still have some limitations, such as low precision and rationality. In order to overcome these limitations, in the light of the theory of set pair analysis and variable fuzzy set, we propose an improved variable fuzzy set pair analysis method (IVFSPA), which combines the analysis framework of variable fuzzy set and set pair analysis, and has made some improvements to the fusion architecture. Firstly, we present a novel game theory comprehensive weighting method, in which the objective entropy method and the subjective analytic hierarchy process(AHP) method employed to obtain the reasonable weight. Then, based on the Nemerow index method, we improve the arithmetic form of “$P_{i}$” (Equation P) to replace the fuzzy comprehensive evaluation method. Furthermore, we design a double judgment mode of combining the principle of maximum membership degree with the positive and negative relationship between the standard value and the measured value, which can accurately judge the evaluation level of surface water quality. Finally, to validate and verify the effectiveness of the proposed method, experiments was conducted at the representative river collection sections of Nanking, China, employing water quality data of 14 sampling sections in their rivers in Nanking during the 2017 flood. In terms of performance metcrics of precision and rationality, based on the values of “TP”, “NH_3_-N”, “Pb”, “AS” and “KMnO_4_” of “Ch-lh section/Chuhe gate” are 0.415, 3.77, 0.07, 0.23 and 7.12, respectively, the level of Ch-lh section/Chuhe gate is that the IVFSPA is Class V and the rest are class IV. Results of experiments show that our IVFSPA method can achieve a good performance, compared with other traditional methods.

## 1. Introduction

Drinking water resources are essential for public health; their quality is a key issue for hydrogeologists and water management practitioners. However, with an increasing deficit on global environmental governance, the chances of extreme weather events, such as typhoons, rainstorms and floods, have increased around the world, and it is not surprising that surface water quality declines during floods. 

In order to protect the valuable surface water resources and safeguard the safety of drinking water for people in the flood areas, a comprehensive evaluation of contaminated water quality when surface water is subject to complex pollution such as heavy metals, is becoming a necessity. Therefore, how to improve the accuracy and reliability of the assessment to contaminated surface waters has increasingly attracted researchers’ and environmental managers’ attentions.

Many methods have been developed attempting to evaluate polluted surface waters. Among them, the water quality index (WQI or NSFWQI), which was developed by the National Sanitation Foundation (NSF) [1], has been widely used to characterize surface water. The WQI is obtained by adding the multiplication of the respective weight factor by an appropriated quality-value for each parameter [2]. Based on the WQI, many advanced WQIs have been proposed, such as the entropy weighted water quality index (EWQI), the minimum WQI (WQI_min_) [3], the River Pollution Index (RPI), the Non-Treated Loads index (NTLI) [4], the Total Pollutant Reduction index (TPRI) [5] and the classic ICAUCA index. They have all been widely used as tools for water quality evaluation and management because they can evaluate water quality in a specific area or season quickly and easily [6,7,8,9,10,11,12,13,14,15,16,17,18]. However, they have several drawbacks, such as a wrong decision might be taken as the methods are dependent on the weight given to different parameters. For that reason, there is a limitation that they cannot measure fuzziness environment in the water quality system, which is caused by the vagueness of classification criteria and the imprecision of boundary values among different classes [19,20].

In recent years, several fuzzy methods [21] have been proposed based on fuzzy concept for contaminated surface water evaluation, such as fuzzy synthetic evaluation (FSE) [22,23,24,25], fuzzy inference system (FIS) [2,26,27,28], and fuzzy similarity measure (FSM) [29], because the water quality evaluation of surface water resources is a multi-index and multi-category fuzzy concept. However, the reasoning criteria for FSE, FIS and FSM are point forms. Therefore, when the actual evaluation criteria are adjusted from one point to another to adapt to the three models, there will be limitations [30]. Reference [31] proposed the variable fuzzy set (VFS) concept to solve the problem of fuzziness and uncertainty in the evaluation. Compared with the traditional fuzzy mathematics method, the VFS method can make the evaluation result more reliable [20,32,33,34,35]. The establishment of relative membership functions depends on physical analysis, expert instinct or experience based on different issues [36].

One well-known modified uncertainty theory is set pair analysis (SPA), which uses a single judgment mode. Under certain conditions, it can analyze the degree of connectivity of set pairs, including “same, different, opposite” degree [37,38]. At present, the integrated combination of SPA and VFS (SPA-VFS model) has been successfully applied to surface water quality evaluation [39,40,41,42]. The SPA-VFS model calculates weights by either the subjective AHP method or the objective entropy method, which means the scientific character of weight value is unreasonable for polluted water assessment in complex environment. As a result, the above methods also have some drawbacks, such as low accuracy, for the evaluation of contaminated surface water in complex hydrological environment. In addition, based on theory of assessment of ecological water and method for monitoring and assessment of drinking water [43,44], the metrics of precision, robustness, rationality and versatility that are included in methods are of considerable importance for the high quality of drinking water and can greatly affect human health.

As such, though much efforts have been made for assessment of contaminated surface water, most of them are mainly focused on the basic methods of water quality index, set pair analysis and variable fuzzy sets. To the best of author’s knowledge, few scholars research on constructing comprehensive weight parameters of variable index, and considering the optimized judgment method by improving arithmetic form and double judgment mode. Therefore, the purpose of this paper is to present a novel method of improved variable fuzzy set pair analysis (IVFSPA) in view of the theory of set pair analysis and variable fuzzy set, and design an optimized fusion architecture for assessment of polluted surface water in complex hydrological environment.

The contributions of this work are summarized under three aspects as follows: (1) We develop an improved Nemerow index method by improving the arithmetic form of $P_{i}$ to replace the P-value calculation method of the fuzzy comprehensive assessment method for making the IVFSPA method more prominent and balanced. (2) We design a new game comprehensive weight ideal by employing both objective entropy method and subjective AHP method to optimize the weight parameters (3) We devise a double judgment mode, which fuses the principle of maximum membership degree and the positive and negative relationship between the monitored value and the criterion value, to improve the accuracy and reliability of assessment.

The remainder of this paper is organized as follows: Section 2 presents our proposed IVFSPA method and related knowledge of the IVFSPA. Section 3 presents some experiments undertaken in major river sections of Nanking. Section 4 gives the experimental results and analysis. Finally, concluding remarks are given in Section 5.

## 2. Methods and Materials

In this study, we propose a novel method in surface water quality evaluation based on improved variable fuzzy set pair analysis (IVFSPA), and then make some efficient improvements to the evaluation fusion architecture [40,41,42]. The key steps of IVFSPA method can be simplified as below:

*Step 1*: A set pair analysis is constructed. This is to analyze the characteristics of the two set pairs discussed in the context of certain problems, and to measure and characterize them. Then, by designing the complete information game thinking to optimize the game theory comprehensive weighting method.

*Step 2*: Development of a variable fuzzy set is peerformed. Calculated by the variable fuzzy set evaluation model, the nonnormalized comprehensive relative membership degree of u′i is obtained.

*Step 3*: The improved Nemerow method is constructed. The Nemerow index method is improved to give the arithmetic form of $P_{i}=\sqrt{\left({\bar{P}}_{max1}^{2}+{\bar{P}}_{max2}^{2}+{\bar{P}}_{max3}^{2}+{\bar{P}}^{2}\right)/2}.$

*Step 4*: The improved variable fuzzy set pair analysis method is applied. Aggregated by the analysis frame work of variable fuzzy set and set pair analysis with some innovation and targeted choices for the fusion architecture, the IVFSPA method is applied to judge the evaluation level of surface water quality.

### 2.1. Development of Set Pair Analysis

The set pair analysis method, which combines certainty analysis and uncertainty analysis, was first proposed by Zhao [17,29]. The basic idea is to make the relationship between the deterministic connection and the uncertainty between the objective things being studied as a certain-uncertain system for analysis. The operation process is to analyze the characteristics of the two set pairs discussed in the context of certain problems, and to measure and characterize them, then get the connection degree of “same, different, opposite” of set pair analysis.

#### 2.1.1. Calculation of Connection Degree

In this section, we employ the cosine function to calculate the connection degree *u_ik_* of the measured variable and the grading criterion, and collect the connection degree matrix *Q* according to the equation of *Q =* [*u_ik_*]*_n×p_*.

Based on the classical computational model: *u = a + b I + c J*, domestic and foreign scholars have carried out in-depth research on the calculation model of the connection degree, and made various improvements and innovations. Firstly, the category of “different” concept in “same, different, opposite” is expanded into k degree levels, forming a total of k + 2 evaluation levels. The calculation model of the connection degree can be written as:

(1) $$\mu_{P_{n}-Q_{m}}=\overset{L}{\underset{n=1}{\sum }}w_{n}a_{n}+\overset{K}{\underset{t=1}{\sum }}\overset{L}{\underset{n=1}{\sum }}w_{n}b_{n,t}i_{t}+\overset{L}{\underset{n=1}{\sum }}w_{n}c_{n}j$$

Note that *b_t_* = 1, 2, …, *k*. Secondly, according to the ambiguity of the standard boundary value of the evaluation index level, the operation expression is refined into two forms of a positive indicator and a negative indicator.

**Figure 1:**
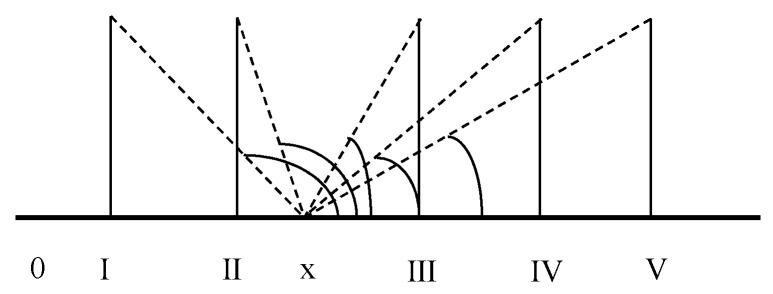
The measurement schematic map.

In view of the characteristics of the components contained in river water and the characteristics of its evaluation in this field, we use coordinate projection method to calculate the connection degree between variable and level criteria. Compared with other computational models, the model has the meaning of image, direct transformation, accurate measurement, and can concisely reflect the actual relationship among variables in connection degree. Refer to the <Surface Water Environmental Quality Standard> (GB3838-2012), when the water quality assessment criteria are set to I, II, III, IV and V levels, the standard value and index values at the same level are on the same axis, and the standard value of each level is set to be the projection of the point directly above one standard unit of each standard value (Figure 1); then, the degree of connection between the index value and the standard value of each level will be shown in a different metrics function. This can be demonstrated from Figure 1.

It can be seen from the analysis that when *a* = 90°, the index value is equal to the standard value, then the degree of connection between the two is 1; and when *a* ≠ 90°, then the degree of connection is a real number between 0 and 1. Based on the set pair analysis theory, combined with the above assumptions and rules, the icon of a metrics is transformed into a functional form of the degree of connection. The latter can be determined according to the equation below:

(2) $$\mu_{ik}=1-\left|\cos a_{ik}\right|=1-\left|\frac{S_{ik}\;-\;x_{i}}{\sqrt{1\;+\;{\left(x_{i}\;-\;S_{ik}\right)}^{2}}}\right|$$

It should be pointed out that although trigonometric functions such as sine, tangent and secant can calculate the degree of connection among model variables, for the simplicity of calculation and the intuitiveness of graphical meaning, we select the cosine function method to solve the degree of connection. In Equation (2), the variable is positioned at level k, and the angle value of index I is *a_ik_*. The measured value is represented by *x_i_*. *S_ik_* is the standard level value, and u_ik_ represents the correlation between index I and evaluation level k.

Based on the values of the degree of connection μ, the values of the connection degree of the “*p*” levels corresponding to the “*n*” indicators of each evaluation sample are constructed in a matrix form and defined as the degree of connection matrix *Q* as shown below:

(3) $$Q={\left[u_{ik}\right]}_{n\times p}=[\begin{array}{cccc} u_{11} & u_{12} & \ldots & u_{1p}\\ u_{21} & u_{21} & \ldots & u_{2p}\\ \ldots & \ldots & \ldots & \ldots \\ u_{n1} & u_{n2} & \ldots & u_{np} \end{array}]$$

#### 2.1.2. Proposed Weighting Method

In this section, we design the complete information game thinking to optimize the game theory comprehensive weighting method. The weights are calculated by the objective entropy method and the subjective AHP method comprehensively, namely: $w=\overset{m}{\underset{i=1}{\sum }}w_{i}w_{ij}$, by which the reasonable weight “*w*” is obtained.

The weight of the index directly affects the comprehensive evaluation results of multiple indicators, and the evaluation results obtained using different indicator weights have large deviations. Therefore, it is important to scientifically assign the indicators to the weight of the facts. The weights generated by various weighting methods are quite different for the evaluation results obtained by the corresponding operations [16]. Thus, the appropriate category of weighting method should be selected according to different situations.

Considering the objectivity of the chemical element properties in the river water and the subjective nature of the expert experience, all of them play a decisive role in the assignment of the variable index of the water sample. Therefore, in order to find a reasonable method suitable for assigning values to the river water variable index, to meet the precise requirements of water quality assessment, we design an empowerment algorithm that combines objective weighting method with subjective weighting method.

The operation process of proposed fusion algorithm is as follows: (1) The entropy method and the AHP are applied to calculate weights of each indicator separately. Equation (4) is an entropy method that represents objective weighting; Equation (5) is an AHP method that represents subjective weighting; (2) By using the complete information game thinking, the above two kinds of weights are calculated by the arithmetic mean method with coefficients; (3) By using comprehensive calculation, a relatively balanced comprehensive weight can be obtained. Then, we devise the main steps of comprehensive weighting based on game theory in a framework, as shown in Algorithm 1.

**Algorithm 1:** Empowerment algorithm for balanced comprehensive weight*Step 1*: Calculated by the entropy method formula, the index weight $w^{1}$ is obtained:
(4)$$w^{1}=\frac{1-f_{k}}{\sum_{k=1}^{m}\left(1-f_{k}\right)}$$
*Step 2*: According to the AHP method, the index weight $w^{2}$ is calculated:
(5)$$w^{2}=\overset{m}{\underset{i=1}{\sum }}w_{i}w_{ij}$$
  The basic weight set determined by the above method is $w_{1}=\left\{w_{11},\;w_{12},\;\ldots ,w_{1m}\right\};{\;w}_{2}$ = {$w_{21}$, $w_{22}$, …, $w_{2m}$}. By arbitrarily linearly combining several weight vectors of the m group, a possible weight vector w can be constructed:
(6)$$w=\sum_{i=1}^{m}\alpha_{i}w_{i}^{T};\;\alpha_{i}>0$$
Note that, *α_i_* is a linear combination coefficient; *m* is the number of groups determining the index weight method. *Step 3*: By optimizing the *i* linear combination coefficients *α_i_* in Equation (7), the deviation of *w_i_* between *w_j_* and each basic weight is minimized. The optimization model of weights is as follows:
(7)$$\min\|\sum_{i=1}^{m}\alpha_{i}w_{i}{}^{T}-w_{j}{}^{T}{\|}_{2};\;j=1,2,\;\ldots ,\;m$$
*Step 4*: According to the differential nature of the matrix, the optimal first derivative condition is obtained:
(8)$$\left[\begin{array}{cccc} w_{1}w_{1}{}^{T} & w_{1}w_{2}{}^{T} & \ldots & w_{1}w_{m}{}^{T}\\ w_{2}w_{1}{}^{T} & w_{2}w_{2}{}^{T} & \ldots & w_{2}w_{m}{}^{T}\\ \ldots & \ldots & \ldots & \ldots \\ w_{m}w_{1}{}^{T} & w_{m}w_{2}{}^{T} & \ldots & w_{m}w_{m}{}^{T} \end{array}\right]\left[\begin{array}{c} \alpha_{1}\\ \alpha_{2}\\ \ldots \\ \alpha_{m} \end{array}\right]=\left[\begin{array}{c} w_{1}w_{1}{}^{T}\\ w_{2}w_{2}{}^{T}\\ \ldots \\ w_{m}w_{m}{}^{T} \end{array}\right]$$
*Step 5*: Solve the equations (*α*_1_, *α*_2_, ..., *α_m_*) by Equation (8), then normalize the coefficient *α_i_*, andthen substitute it into Equation (6) to find the optimal comprehensive weight “*w*”.

The key to optimizing the weight assignment is to determine the optimal composite weight “*w*” from the possible weights. The core idea of using game theory to determine “*w*” is to find this equilibrium among different weights.

### 2.2. Development of Variable Fuzzy Set

In this section, we calculate the nonnormalized comprehensive relative membership degree of *u′_i_*, namely: u′_i_ = 1/ (1 + *d_hg_/d_hb_*) *α*, via the variable fuzzy set evaluation model.

The variable fuzzy set theory is a system of variable fuzzy sets based on engineering fuzzy set theory [20]. Let the domain U be the collection of all surface water samples in the study area, and the fuzzy concept $\tilde{A}$ denotes the classification level of a water sample in the <Surface Water Quality Standard> [17]. The opposite fuzzy concept on U is an arbitrary element *u* (*u*∈U) in U, which is a point on the continuous number axis of the relative membership function. The relative membership degree of “*u*” to the attraction property $\tilde{A}$ is $\mu_{\tilde{A}}\left(u\right)$; the relative membership degree to the rejection property $\tilde{A_{c}}$ is $\mu_{\tilde{A_{c}}}\left(u\right)$. where $\mu_{\tilde{A}}\left(u\right),u\in \left[0,1\right]$; $\mu_{\tilde{A_{c}}}\left(u\right),\left(u\in \left[0,1\right]\right)$.

Assuming that $D_{\tilde{A}}\left(u\right)=\mu_{\tilde{A}}\left(u\right)-\mu_{\tilde{A_{c}}}\left(u\right)$, then $D_{\tilde{A}}\left(u\right)$ is called the relative difference of “*u*” to $\tilde{A}$; and then the relative difference function of “*u*” to $\tilde{A}$ is expressed below:

(9) $$\{\begin{array}{c} D_{\tilde{A}}:D\to \left[-1,1\right]\\ u|\to D_{\tilde{A}}\left(u\right)\in \left[-1,1\right] \end{array}\;\;;\;\mu_{\tilde{A}}\left(u\right)+\mu_{\tilde{A_{c}}}\left(u\right)=1$$

**Figure 2:**
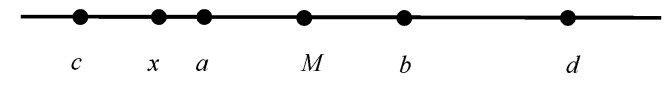
The sketch map of location.

Let *X_0_* = [a, b] be the attracting domain of the fuzzy variable set $\tilde{V}$ on the real axis, and *X* = [c, d] be the interval of a certain upper and lower bound range containing *X*_0_ (*X*_0_ ⊂ *X*), which is called exclusion domain (Figure 2). It can be demonstrated in Figure 2.

The relative difference function model when “*x*” falls to the left of “*M*” point could be put as below when “*M*” is the point value of $D_{\tilde{A}}\left(u\right)$ = 1 in the attraction domain interval [a,b], in the meanwhile, “*x*” is the measured value of any point in the X interval:

(10) $$D_{\tilde{A}}\left(u\right)={\left(\frac{x-a}{M-a}\right)}^{\beta },x\in \left[a,M\right];\;D_{\tilde{A}}\left(u\right)={\left(\frac{x-a}{c-a}\right)}^{\beta },\;x\in \left[c,a\right]$$

When “*x*” falls to the right of point M, the relative difference function model is:

(11) $$D_{\tilde{A}}\left(u\right)={\left(\frac{x-b}{M-b}\right)}^{\beta },x\in \left[M,b\right];\;D_{\tilde{A}}\left(u\right)=-{\left(\frac{x-b}{d-b}\right)}^{\beta },\;x\in \left[b,d\right]$$

Note that, “*β*” is a non-negative index, such as β = 1. When the number of evaluation indicators is “*t*” and the evaluation level is “*c*”, then the variable fuzzy set evaluation model is:

(12) $$u_{h}^{'}=\frac{1}{\left[1+{\left(\frac{d_{hg}}{d_{hb}}\right)}^{\alpha }\right]};\;h=1,\;2,\;\ldots ,\;c$$

(13) dhg={∑i=1t[wi(1−μA˜(uih))]p}1p

(14) dhb=∑i=1t[μA˜wi−(uih)p]1p

It should be noted that $u_{h}^{\prime }$ is the non-standardized comprehensive relative membership of the sample relative to the “*h*” level, where “*p*” is the distance parameter. When *p* = 1, it is a linear model, and when *p* = 2, it is a nonlinear model. “α” is the optimization standard parameter, usually *α* = 1 or *α* = 2. “*w_i_*” is the comprehensive weight of the *i*-th evaluation index.

### 2.3. Proposed Improved N.L.Nemerow Method

In this section, the Nemerow index method [34,35] is improved to form the arithmetic form of $P_{i}=\sqrt{\left({\bar{P}}_{max1}^{2}+{\bar{P}}_{max2}^{2}+{\bar{P}}_{max3}^{2}+{\bar{P}}^{2}\right)/2}.$ The proposed improved Nemerow method replaces the “*P*” value calculation method of the fuzzy comprehensive evaluation method with “*P_i_*”, making the IVFSPA more prominent and balanced for evaluation of contaminated surface water quality.

In view of the fact that the fuzzy comprehensive evaluation method is prone to repeated calculation of evaluation indicators, information loss and the effect of weights are not significant, we develop the improved Nemerow method to improve the assessment of surface water. The core idea of the Nemerow method is the transformation of different indicators of the mean value of the evaluation ratio. The conversion formula can be written as:

(15) $$P_{1}=\sqrt{\left(P_{max}^{2}+{\bar{P}}^{2}\right)/2}$$

(16) $$P_{i}=\frac{c_{i}}{s_{ij}};\;I=1,\;2,\ldots ,\;n;\;j=1,\;2,\;\ldots ,\;m$$

where *P_i_* is the Nemerow pollution index. *C_i_* is the gauged concentration of the *i*-th assessment factor; *S_ij_* is the *j*-th standard concentration of the *i*-th assessment factor; *P_max_* is the maximum value of *P_i_*; $\bar{P\;}$ is the average value of *P_i_*.

However, after a river becomes polluted, the requirements for its assessment are very strict and the evaluation is very difficult. In addition, it is found by analysis of Equation (15) that the algorithm overemphasizes the influence of the maximum pollution factor on water pollution. In fact, there are usually numerous special evaluation factors, such as TP, NH_3_-N, Pb, As, KMnO_4_, whose measured concentrations are not large, but have a big negative influence on water quality, and that this bad influence has not been well reflected only by their weights. Therefore, it is necessary to improve the traditional Nemerow method.

Based on a large number of studies by domestic and foreign scholars, we have carried out some improvement and innovative ideas for the Nemerow method as follows. The first is to replace $P_{max}^{2}$ with ${\bar{P}}_{max}^{2}$ to constrain the role of the maximum influence factor. The second is to increase the operational elements of ${\bar{P}}_{max2}^{2}$ and ${\bar{P}}_{max3}^{2}$ to highlight the huge role of special variables and take into account multidimensional variable indicators. The third is to calculate the arithmetic mean of *P_i_* and *P_max_*_1_, *P_max_*_2_, *P_max_*_3_, and assign them to ${\bar{P}}_{max1}^{2}$, ${\bar{\;P}}_{max2}^{2}\;$ and ${\bar{P}}_{max3}^{2}$. The improved calculation formula can be written as:

(17) $$P_{2}=\sqrt{\left({\bar{P}}_{max1}^{2}+{\bar{P}}_{max2}^{2}+{\bar{P}}_{max3}^{2}+{\bar{P}}^{2}\right)/2}$$

where *P_2_* is the improved Nemerow pollution index; $\bar{P}max1$ is the arithmetic mean of the *P_i_* value corresponding to the maximum weight factor and *P_max_*_1_; the corresponding value of $\bar{P}max2$ is the *P_i_* value corresponding to the second largest factor of weight and *P_max2_*; $\bar{P}max3$ is the arithmetic mean of the third largest *P_i_* value and *P_max_*_3_.

Obviously, the improved Nemerow method takes into account the weight of each pollution factor in water quality assessment, and weakens the impact of the largest pollution factor on water quality. In particular, compared with the traditional fuzzy synthetic operator operation (Table 1), it shows obvious prominence and balance.

### 2.4. The Improved Variable Fuzzy Set Pair Analysis Method

In the following, we propose an improved variable fuzzy set pair analysis method aggregated by the analysis frame work of variable fuzzy set and set pair analysis with some innovation and targeted choices for the fusion architecture.

Firstly, in view of the limitations of the four synthetic operations of fuzzy comprehensive evaluation, we design the weighted idea of the improved Nemerow method to optimize the algorithm of traditional synthetic operators. Combined with the advantages of optimized traditional methods [14,37], a new surface water quality evaluation model, namely the IVFSPA model, was built. The calculation formula of the IVFSPA model is shown below:

(18) $$C=W⊗Q=\left[\begin{array}{cccc} w_{1}, & w_{2}, & \ldots , & w_{m} \end{array}\right]⊗\left[\begin{array}{cccc} u_{11}^{\prime } & u_{12}^{\prime } & \ldots & u_{1p}^{\prime }\\ u_{21}^{\prime } & u_{21}^{\prime } & \ldots & u_{2p}^{\prime }\\ \ldots & \ldots & \ldots & \ldots \\ u_{n1}^{\prime } & u_{n2}^{\prime } & \ldots & u_{np}^{\prime } \end{array}\right]$$

Secondly, we employ the proposed improved Nemerow index idea to replace the relative degree of difference *u′_i_* of the corresponding unified level with the connection degree *u_i_* of each evaluation level, and then complete the calculation of C = (*p*_1_, *p*_2_, ..., *p_p_*).

In the proposed IVFSPA model, the idea of the variable fuzzy set is adopted, and the connection degree $u_{i}$ of each evaluation level is substituted for the relative difference degree $u_{i}^{\prime }$ of the corresponding unified level. The equation can be expressed as follows:

(19) $$u_{ip}=\alpha_{i}u_{ip}^{\prime }=\alpha_{i}\frac{1}{\left[1+{\left(\frac{d_{ig}}{d_{ib}}\right)}^{\alpha }\right]}$$

where *α_i_* is the optimization parameter of the *i*-th evaluation level.

Then, Equation (19) is substituted into Equation (18), and the conversion is obtained as below:

(20) $$\begin{array}{c} C=\left[\begin{array}{cccc} w_{1}, & w_{2}, & \ldots , & w_{m} \end{array}\right]⊗\left[\begin{array}{cccc} u_{11}^{} & u_{12}^{} & \ldots & u_{1p}^{}\\ u_{21}^{} & u_{21}^{} & \ldots & u_{2p}^{}\\ \ldots & \ldots & \ldots & \ldots \\ u_{n1}^{} & u_{n2}^{} & \ldots & u_{np}^{} \end{array}\right]=\left[\begin{array}{cccc} w_{1}u_{11} & w_{1}u_{12} & \ldots & w_{1}u_{1p}\\ w_{2}u_{21} & w_{2}u_{21} & \ldots & w_{2}u_{2p}\\ \ldots & \ldots & \ldots & \ldots \\ w_{n}u_{n1} & w_{n}u_{n2} & \ldots & w_{n}u_{np} \end{array}\right]\\ =\left[\begin{array}{cccc} P_{11} & P_{12} & \ldots & P_{1p}\\ P_{21} & P_{21} & \ldots & P_{2p}\\ \ldots & \ldots & \ldots & \ldots \\ P_{n1} & P_{n2} & \ldots & P_{np} \end{array}\right] \end{array}$$

where the symbol “⊗”is the improved synthesis operator.

Based on the idea of the improved Nemerow method, let:

(21) $$\begin{array}{l} C=(p_{1},p_{2},\ldots ,p_{p})=\\ \left(\sqrt{{\bar{P}}_{{}_{1\max1}}^{2}+{\bar{P}}_{{}_{1\max2}}^{2}+{\bar{P}}_{{}_{1\max3}}^{2}+{\bar{P}}_{1}^{2}},\sqrt{{\bar{P}}_{{}_{2\max1}}^{2}+{\bar{P}}_{{}_{2\max2}}^{2}+{\bar{P}}_{{}_{2\max3}}^{2}+{\bar{P}}_{2}^{2}},\ldots ,\sqrt{{\bar{P}}_{{}_{p\max1}}^{2}+{\bar{P}}_{{}_{p\max2}}^{2}+{\bar{P}}_{{}_{p\max3}}^{2}+{\bar{P}}_{p}^{2}}\right) \end{array}$$

It is worth noting that ${\bar{P}}_{imax1}$, ${\bar{P}}_{imax2}$ and ${\bar{P}}_{imax3}\;$ represent the three average values of the maximum values in *P*_1*i*_, *P*_2*i*_,…, *P_ni_*, respectively, and the product of the first three indicators with the largest weight. $\bar{P_{i}}$ is the average of *P*_1*i*_, *P*_2*i*_,…, *P_ni_*; *i* = 1, 2, ..., *p*.

Although according to the principle of maximum connection, when *p_j_* = max (*p_1_, p_2_, ..., p_p_*), then, it can be determined that the water quality level of the river is the “*j*” level. In order to accurately evaluate the level of river water quality, we set the following further research and judgment on the water quality of the river section. In this case, instead of fixing the water quality to the class *j*-th simply, we located the river water quality assessment level between the *j*-th and *j* + 1-th levels.

Finally, we devise the double judgment mode with the principle of maximum membership degree and the positive and negative relationship between the monitored value and the standard value to accurately determine the evaluation level of surface water quality.

With the help of Figure 2, we set *M_ik_* as the marker variable to represent the positive and negative relationship of the measured value with respect to the standard value. When the corresponding angle “*a*” in the figure satisfies *a* ≥ 90°, then:

(22) $$M_{ik}=u_{ik};else,M_{ik}=-u_{ik}$$

and we set:

(23) $$M_{j}=\sum_{i=1}^{n}w_{i}M_{ij},$$

Then, the surface water quality level judgment has the following two conditions: (1) When *M_j_* < 0, then the comprehensive connection number of the evaluation index is in the negative direction of the *j*-th level criterion value; accordingly, the surface water quality level is judged as j+1 level. (2) When *M_j_* > 0, then the comprehensive connection number of the evaluation index is in the positive direction of the *j*-th level criterion value; accordingly, the water quality of the measured river section is located as the *j*-th level.

### 2.5. Study Area and Sampling

Nanking, located on the eastern coast of China, with a developed water system and abundant precipitation, was selected as an example for this study. The area and average altitude are approximately 6587 km^2^ and 20 MSL, respectively. The average annual rainfall is 117 days, the average rainfall is 1106.5 mm, and the relative humidity is 76%. According to the Emberger climate classification, the area is under the influence of a subtropical humid climate with four distinct seasons and abundant rain. Nanjing has a rich plant resources and a wide variety of plants, with a forest coverage rate of 29.6%. The area belongs to the hilly area. The low mountains account for 3.5% of the total land area, the hills account for 53%, and the total area of plains, depressions and rivers and lakes accounted for 39.2%. The water area is over 11% of the total area. There are 120 large and small rivers in the territory, which can be divided into four major water systems of the Yangtze River Nanjing section, the Weihe River, the Qinhuai River and the Qingyi River and Shuiyang River.

**Figure 3:**
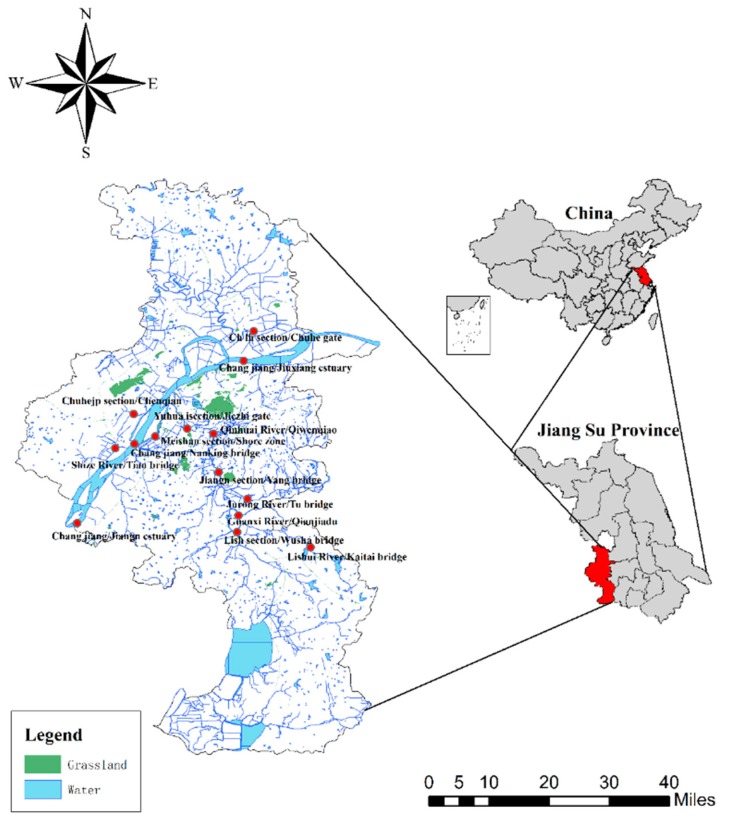
Geology and geographic location of the research area.

It is one of the three core cities in the “Yangtze River Delta” region, and is connected to Beijing and Shanghai. The geographical location and strategic position are very important (Figure 3). Therefore, maintaining the safety of surface water resources in the region is of great significance to safeguarding national economic development and social stability. In this study, we select the representative river collection section of Nanking, such as Qinhuai River/Qiqiaowen, Guanxi River/Qianjiadu,Chuhe jp section/Chenqian, Chuhe lh section/Chuhe gate, Yuhuai section/Jiezhi gate, Jiangning section/Yang bridge, Lishui section/Wusha bridge, Jurong River/Tu bridge, Chang jiang/Nanking bridge, Lishui River/Kaitai bridge, Shize River/Tian bridge, Chang jiang/Jiangning estuary, Chang jiang/Jiuxiang estuary, Meishan section/shore zone, as a surface water sample collection point to collect sample data (Table 2) to comprehensively reflect the overall situation of surface water quality in Nanking.

### 2.6. Evaluation Indicators and Class Criteria

In this study, considering that Nanking has a high degree of industrialization, especially light industry, its surface hydrological environment has corresponding regional and special characteristics. Moreover, considering that the dimension of the variables needed to assess the surface water level, we select “TP”, “NH_3_-N”, “Pb”, “As”, “KMnO_4_”, “FC”, “DO”, “COD” and “BOD_5_” as the main indicators for water quality evaluation [45,46,47], combined with the actual characteristics of the surface hydrological environment in Nanking. The different class values corresponding to the evaluation indicators (Table 3) are shown as follows.

## 3. Experiments

In this section, we take the data collected from representative rivers in Nanking as samples in order to evaluate the effectiveness of our proposed IVFSPA method for assessment of polluted surface water. The simulation operation framework of IVFSPA method can be summarized as follows:

*Step 1*: Taking Qinhuai River/Qiqiaowen as an example, we collect the source data of the river section collection point (for example, TP = 0.417), and calculate the degree of connection using Equations (2) and (3) in Section 2.1 [20,32]. They can be expressed in the form of a matrix as below:

(24) $$\mu_{11}=1-\left|\cos a_{11}\right|=1-\left|\frac{S_{ik}-x_{1}}{\sqrt{1+{\left(x_{1}-S_{11}\right)}^{2}}}\right|$$

Then, the degree of connection between the indicator “TP” and the assessment level “I” can be computed as follows:

(25) $$\mu_{11}=1-\left|\frac{0.02\;-\;0.417}{\sqrt{1\;+\;{\left(0.417-0.02\right)}^{2}}}\right|=1-0.3429=0.657$$

In the same way, the five levels of connection corresponding to the nine indicators of “Qinhuai River/Qiqiaowen” are obtained, and expressed as: $\mu_{1,2},\mu_{1,3},\ldots ,\mu_{9,5}$. The total connection degree of the assessment index of the surface water collection point is collected and constructed into a 9 × 5 degree of connection matrix *Q*. The numerical values in the form of the evaluation index *Q* matrix are shown below.

(26) $$Q=u_{9\times 5}=\left[\begin{array}{ccccc} 0.6571 & 0.7119 & 0.7928 & 0.8846 & 0.9830\\ 0.7300 & 0.7047 & 0.6611 & 0.6071 & 0.3590\\ 0.8817 & 0.8817 & 0.9205 & 0.9205 & 0.9700\\ 0.5094 & 0.5166 & 0.5166 & 0.5247 & 0.5247\\ 0.7891 & 0.6572 & 0.5907 & 0.7346 & 0.8837\\ 0.7346 & 0.7225 & 0.6552 & 0.5385 & 0.600\\ 0.7631 & 0.6522 & 0.5349 & 0.5862 & 0.5420\\ 0.8750 & 0.8750 & 0.6893 & 0.8625 & 0.9418\\ 0.5131 & 0.5131 & 0.7565 & 0.5680 & 0.8809 \end{array}\right]$$

*Step 2*: Calculate the weight value of the water quality assessment index according to the Equations (4) to (5) in Section 2.1 as below [16]:

(27) $$w^{1}=\left(0.3375\;\;0.3783\;\;0.3289\;\;0.3224\;\;0.3391\;\;0.1809\;\;0.2657\;\;0.2332\;\;0.3352\right)$$

(28) $$w^{2}=\left(0.2067\;\;0.2863\;\;0.1681\;\;0.1655\;\;0.2147\;\;0.0736\;\;0.1193\;\;0.1011\;\;0.1723\right)$$

Obviously, there are some differences in the weight values of $w^{1}$ and $w^{2}$. That fact further illustrates the necessity and rationality of using the game theory comprehensive weighting method proposed in this study.

Therefore, the fusion operations of $w^{1}$ and $w^{2}$ are obtained using Equations (6)–(8), and the fusion results are normalized. The comprehensive weight value of the water quality assessment indicator is obtained and represented by “*w*”. The values are obtained as follows:

W = (0.1241 0.1425 0.1218 0.1207 0.1246 0.0696 0.0914 0.0826 0.1227)(29)

*Step 3*: The comprehensive weighted connection degree and its “*P*” matrix “*M*” can be computed by equations from (18) to (20) [34,37]:

C = (0.1241 0.1425 0.1218 0.1207 0.1246 0.0696 0.0914 0.0826 0.1227)

(30) $$⊗\left[\begin{array}{ccccc} 0.6571 & 0.7119 & 0.7928 & 0.8846 & 0.9830\\ 0.7300 & 0.7047 & 0.6611 & 0.6071 & 0.3590\\ 0.8817 & 0.8817 & 0.9205 & 0.9205 & 0.9700\\ 0.5094 & 0.5166 & 0.5166 & 0.5247 & 0.5247\\ 0.7891 & 0.6572 & 0.5907 & 0.7346 & 0.8837\\ 0.7346 & 0.7225 & 0.6552 & 0.5385 & 0.600\\ 0.7631 & 0.6522 & 0.5349 & 0.5862 & 0.5420\\ 0.8750 & 0.8750 & 0.6893 & 0.8625 & 0.9418\\ 0.5131 & 0.5131 & 0.7565 & 0.5680 & 0.8809 \end{array}\right]$$

(31) $$=\left[\begin{array}{ccccc} 0.0815 & 0.0883 & 0.0984 & 0.1098 & 0.1220\\ 0.1040 & 0.1004 & 0.0942 & 0.0865 & 0.0511\\ 0.1074 & 0.1074 & 0.1121 & 0.2007 & 0.1181\\ 0.0615 & 0.0624 & 0.0624 & 0.0633 & 0.0633\\ 0.0983 & 0.0819 & 0.0736 & 0.0915 & 0.1101\\ 0.0511 & 0.0503 & 0.0456 & 0.0375 & 0.0418\\ 0.0697 & 0.0596 & 0.0489 & 0.0536 & 0.0495\\ 0.0723 & 0.0754 & 0.0569 & 0.0712 & 0.0778\\ 0.0630 & 0.0630 & 0.0928 & 0.0697 & 0.1081 \end{array}\right]$$

*Step 4*: According to the idea of the improved Nemerow method, the following results are calculated via Equation (20) [17,40]:

(32) $$\begin{array}{c} p_{1}=\sqrt{{\left(\frac{0.1040+0.1074}{2}\right)}^{2}+{\left(\frac{0.0983+0.1074}{2}\right)}^{2}+{\left(\frac{0.0815+0.1074}{2}\right)}^{2}+{\left(\frac{1}{9}\sum_{n=1}^{9}p_{n,1}\right)}^{2}}=\\ \sqrt{0.0112+0.0106+0.0089+0.0062}=0.1921 \end{array}$$

In the same way, the results of “*P*_2_~*P*_5_” can be computed via the above equation:

(33) $$P_{2}=\sqrt{{\left(\frac{0.1004+0.1074}{2}\right)}^{2}+{\left(\frac{0.0819+0.1074}{2}\right)}^{2}+{\left(\frac{0.0883+0.1074}{2}\right)}^{2}+{\left(\frac{1}{9}\sum_{n=1}^{9}p_{n,2}\right)}^{2}}=0.1879$$

(34) $$P_{3}=\sqrt{{\left(\frac{0.0942+0.1121}{2}\right)}^{2}+{\left(\frac{0.0736+0.1121}{2}\right)}^{2}+{\left(\frac{0.0984+0.1121}{2}\right)}^{2}+{\left(\frac{1}{9}\sum_{n=1}^{9}p_{n,3}\right)}^{2}}=0.1899$$

(35) $$P_{4}=\sqrt{{\left(\frac{0.0865+0.2007}{2}\right)}^{2}+{\left(\frac{0.0915+0.2007}{2}\right)}^{2}+{\left(\frac{0.1098+0.2007}{2}\right)}^{2}+{\left(\frac{1}{9}\sum_{n=1}^{9}p_{n,4}\right)}^{2}}=0.2713$$

(36) $$P_{5}=\sqrt{{\left(\frac{0.0511+0.1220}{2}\right)}^{2}+{\left(\frac{0.1101+0.1220}{2}\right)}^{2}+{\left(\frac{0.1220+0.1220}{2}\right)}^{2}+{\left(\frac{1}{9}\sum_{n=1}^{9}p_{n,5}\right)}^{2}}=0.2066$$

Combining equations from (32) to (36) we get:

(37) $$C=(p_{1},p_{2},\ldots ,p_{p})=(P_{1},P_{2},P_{3},P_{4},P_{5})=(0.1921,0.1879,0.1899,0.2713,0.2066)$$

*Step 5*: Refer to the index relationship map in Section 2.1, and use the principle of maximum membership degree and the double judgment method of the positive and negative relationship between the standard value and the measured value to evaluate the water quality level of the “Qinhuai River/Qiqiaowen” collection point [34,38]. The level is determined according to the value *Pj*. And the value *Pj* can be calculated by the following formula:

*P_j_* = Max (*P*_1_,*P*_2_,*P*_3_,*P*_4_,*P*_5_) (38)

Then, the *Pj* of the “Qinhuai River/Qiqiaowen” collection point can be obtained:

*P_j_* = *P*_4_ (39)

Therefore, it can be judged that the water sample level of the “Qinhuai River/Qiqiaowen” collection point is the largest in connection with the IV level. Hence, the result can be located that the water quality of the “Qinhuai River/Qiqiaowen” collection point is between the IV level and the V level. Further analysis, based on Equations (23) and (38) [37,38], we can easily draw the conclusion below. Because of *P_j_ = P_4_*, we get $M_{j}=M_{4}=\sum_{i=1}^{9}w_{i}M_{i4}=\sum_{i=1}^{9}w_{i}u_{i4}$. Substituting the corresponding weight “$w_{i}$” and the value “$u_{i4}$” into the above function, we obtain *Mj* < 0. Accordingly, it is confirmed that the water quality level of the “Qinhuai River/Qiqiaowen” collection point is class V.

In the same way, we collect the sample data of the remaining 13 water quality assessments such as “Guanxi River/Qianjiadu” and substitute them into the improved variable fuzzy set pair analysis model to obtain the all assessment results of the IVFSPA method (Table 4).

## 4. Results and Discussion

### 4.1. Analysis of Assessment Results

In order to make the result analysis more convincing, in this section, we collect sample data of the same water quality as the IVFSPA, and use 7 different evaluation methods [2,6,7,8,9,10], such as the NSFWQI, to perform the calculation separately. Further, the corresponding evaluation results (Table 5) are obtained as below.

The analysis of the evaluation results of the water samples at different collection points in Table 5 shows that the evaluation level of “Qinhuai River/Qiqiaowen” water quality is that the EWQI and the ICARCA are class IV, and the rest of the methods are class V. The evaluation level of Guanxi River/Qianjiadu is that the NSFWQI and the VFSPA are class IV, and the rest of the methods are Class V. The level of Chuhe-jp section/Chenqian is that the NSFWQI and the TSKFWQI are Class III, and the rest are Class IV. The level of Ch-lh section/Chuhe gate is that the IVFSPA is Class V and the rest are class IV. The level of Yuhuai section/Jiezhi gate is that the ICUACA is class V and the rest are class IV. The level of Jiangn section/Yang bridge is that the TSKFWQI is class V and the rest are class IV. The level of Li-sh section/Wusha bridge and Shize River/Tian bridge are all class V. The level of Jurong River/Tu bridge is that the FSEVFS is class IV and the rest are class V. The level of Chang jiang/Nanking bridge is class V.

The level of Lishui River/Kaitai bridge is that the IVFSPA is class V and the rest are class IV. The level of Chang jiang/ Jiangn estuary is that the EWQI is class III and the rest are class IV. The level of Chang jiang/Jiuxiang estuary is that the ICAUCA is class III and the rest are class IV. The level of Meishan section/shore zone is that the FSEVFS is class IV and the rest are class V.

Obviously, among the above evaluation methods, only the proposed IVFSPA method judged that the water quality of “Ch-lh section/Chuhe gate” and “Lishui River/Kaitai bridge” are class V. The reason for this is that the double judgment mode, adopted by the IVFSPA method, which is combined with the maximum membership degree principle and the positive and negative relationship between the standard value and the measured value, takes into account the balance between the standard values of $M_{j}$ and $M_{j+1}$ and the measured values. Thus, it can avoid the errors caused by a single judgment of the principle of maximum membership. Ultimately, the evaluation results of the IVFSPA method are more accurate and reliable. In addition, from the comprehensive weighted connection degree of “Ch-lh section/Chuhe gate” in Table 4, we can see that its class III value is 0.1783, the class IV is 0.2719, and the class V is 0.2057. Hence, this level is initially positioned between level IV and level V. Through further observation, we found that the value of “*P*_5_” in the matrix is significantly larger than the value of “*P*_3_”. Therefore, the result of the second judgment is class V, which is more scientific and reasonable.

In this section, we provide an in-depth analysis of the evaluation results represented by “Ch-lh section/Chuhe gate” and “Lishui River/Kaitai bridge” from different angles, and obtain a consistent judgment result. At the same time, that result powerfully confirms the progressiveness and rationality of the IVFSPA method.

### 4.2. Method Performance Evaluation

In order to further confirm the superiority of the proposed IVFSPA method, and prove that the method is more accurate and reasonable for the assessment of surface water, we take the evaluation results of item 4 of “Ch-lh section/Chuhe gate” and item 10 of “Lishui River/Kaitai bridge” in Table 4 as an example to further analyze and discuss the actual measured data (Table 2). It is not difficult to find that a common feature of the two is that the total value of the factor indicators, which are “TP” and other factors that seriously pollute the environment, is relatively higher than the total value of other water sample collection points. Where the values of “TP”, “NH_3_-N”, “Pb”, “AS” and “KMnO_4_” of “Ch-lh section/Chuhe gate” are 0.415, 3.77, 0.07, 0.23 and 7.12, respectively. The values of “Lishui River/Kaitai bridge” are 0.421, 3.80, 0.06, 0.46, and 7.02, respectively. Although the measured concentrations of these elements are not large, they have a huge influence to water quality. Moreover, their pollution effects on water quality are more efficient than other indicators.

**Figure 4:**
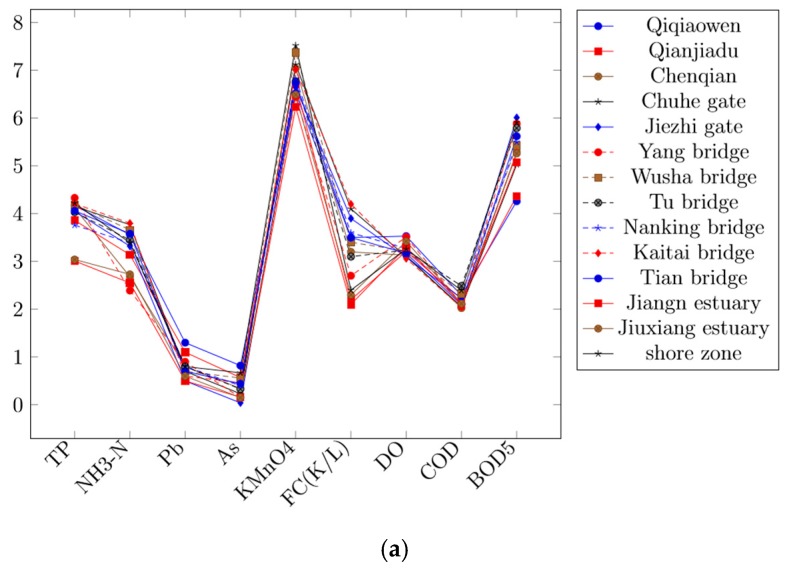

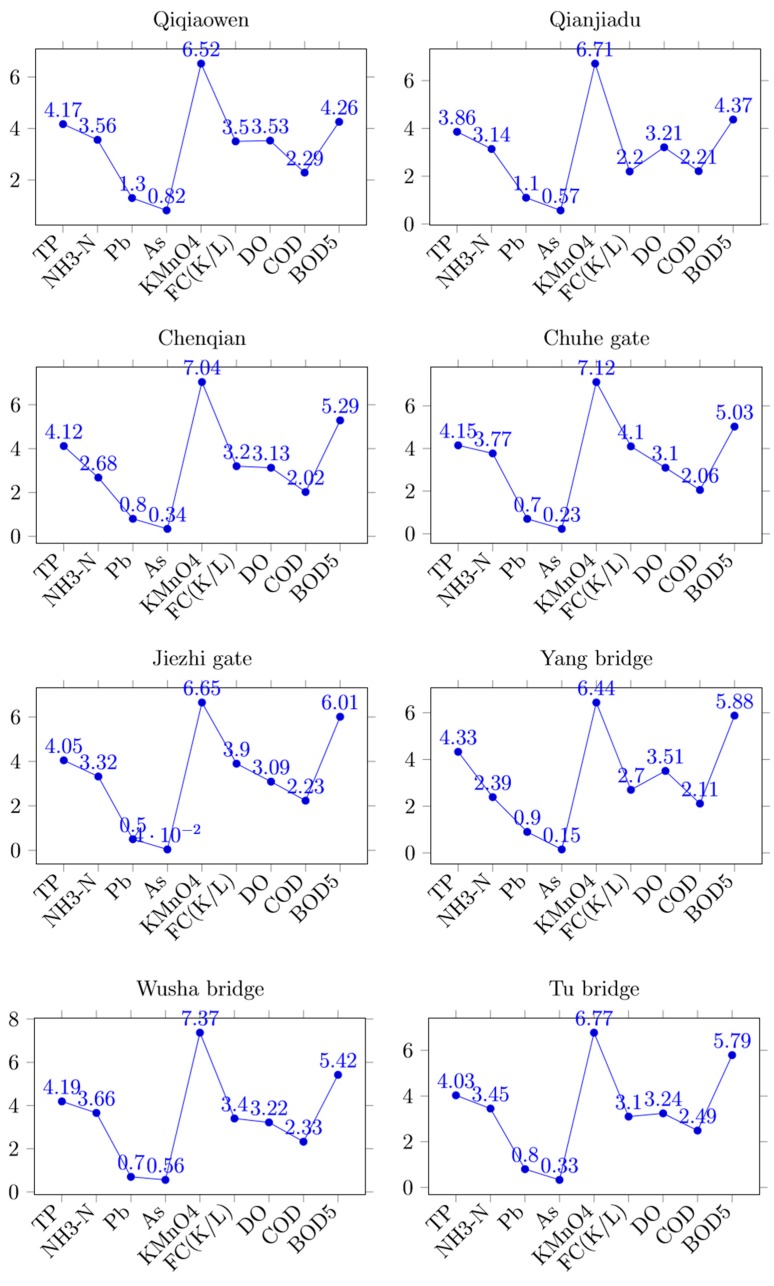

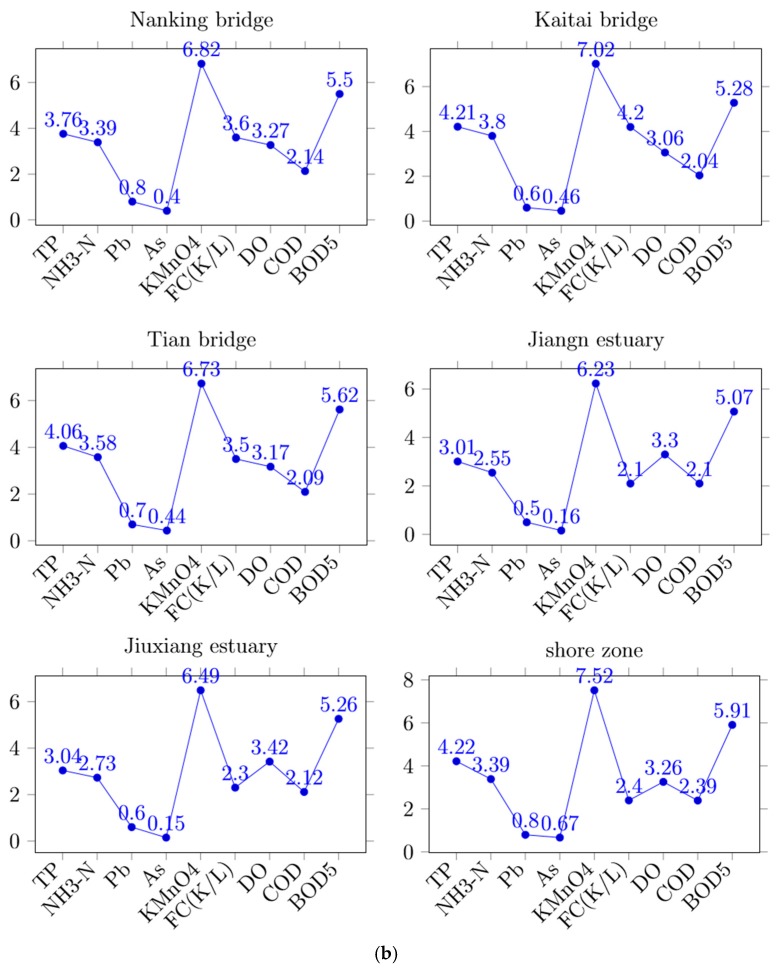
(**a**) River section illustration, (**b**) River section illustration. Note that, since the measured values of different factors have large difference, some measured values are converted into a multiple form to make the contrast effect more obvious.

For a more intuitive comparison and analysis, we convert the sample values of the representative river collection sections in Table 2 into the form of curves (Figure 4). Here, curve 4 represents the factor value of “Ch-lh section/Chuhe gate” and curve 10 represents the factor value of “Lishui River/Kaitai bridge”. Obviously, the numerical curves of “TP”, “NH_3_-N”, “Pb”, “AS” and “KMnO_4_” corresponding to curve 4 and curve 10 are overall at the top of the curve group, which means that the sum of the values of the five factors in the group is relatively high. In addition, the numerical curves corresponding to the above factors of the collection points, which are V level, such as “Qinhuai River/Qiqiaowen”, “Li-sh section/Wusha bridge” and “Meishan section/shore zone” etc., are located overall at the top of the curve group too. Table 2 shows that the sum of their factor values is also relatively larger. It means more seriously contaminated. The illustration of the pollution levels of river sections, such as “Ch-lh section/Chuhe gate” and “Lishui River/Kaitai bridge”, can be demonstrated in Figure 4.

**Figure 5:**
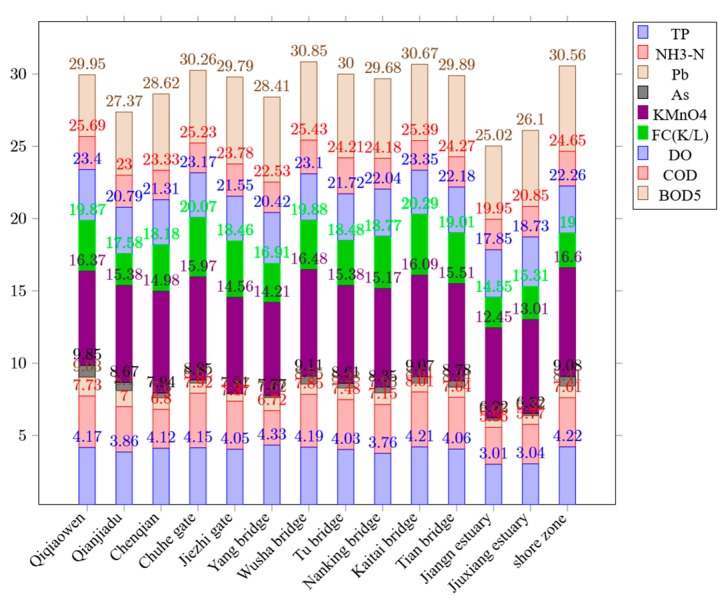
Impact factor illustration.

To more fully verify the importance relationship between the impact factors and assessment levels, we use impact factor illustration; as a result, the same conclusion as the above is obtained (Figure 5). The illustration of the numerical results of the impact factors is displayed in Figure 5.

For that reason, although many researchers had given bigger weight to the influence factors such as “TP” in the traditional evaluation method, the corresponding evaluation index does not fully reflect the influence of the above elements on water quality. The reason is that the value of the variable factor is very weak. Therefore, they have more or less errors in the judgment of water quality levels, and usually appear to be “undervalued”.

However, our proposed IVFSPA method not only uses the game comprehensive weight method and the improved Nemerow method to optimize the weights of every indicator, but also adds the part of double judgment of the positive and negative relationship between the standard value and the measured value [38,40], and finally revises the assessment level of “Ch-lh section/Chuhe gate” and “Lishui River/Kaitai bridge” to level 5. Obviously, this judgment is more accurate and reasonable; and the actual pollution of “Ch-lh section/Chuhe gate” and “Lishui River/Kaitai bridge” also supports that judgment. The IVFSPA method effectively compensates for the defects and shortcomings of the assessment methods proposed by the predecessors on the surface water quality assessment. Besides, it emphasizes the huge role played by pollution factors such as “TP” in water quality assessment, mitigates the negative impact of maximum pollution factors on water quality, and takes into account the role of general pollution factors. Then, in the evaluation application, the advanced and balanced characteristics of the surface water quality assessment by the IVFSPA method are shown.

### 4.3. Metrics Validation of Method Performance

Although it is very difficult to validate the proposed method by metrics, validation can be attempted through four distinct aspects. Based on the actual situation of the contaminated water, we employ the same numerical simulation method as the above, and collect samples from 14 sampling sections of representative rivers in Nanking, then carry out the same evaluation experiment over 1000 times. The experiment results of metrics verification of the seven various methods, such as NSFWQI, EWQI, ICAUCA, TSKFWQI, FSEVFS, VFSPA and IVFSPA [2,6,7,8,9,10], are as follows.

**Figure 6:**
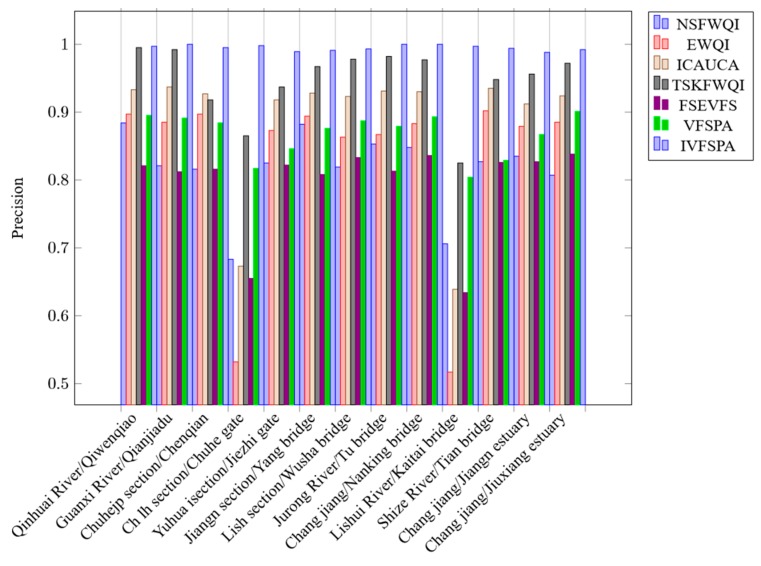
Precision values.

In Figure 6, the horizon axis represents the river collection sections names of Nanking, and the vertical axis represents the values of precision. Of all experiments, the proposed IVFSPA method has a good ability of precision, which achieves the highest precision value (0.998) of all methods. Meanwhile, we can see that other methods like NSFWQI, EWQI, ICAUCA, TSKFWQI, FSEVFS and VFSPA have not obtain a higher level of precision compared with IVFSPA methods (have some weakness in precision assessment). Here, the precision values of them are 0.884, 0.902, 0.939, 0.995, 0.838 and 0.901, respectively.

**Figure 7:**
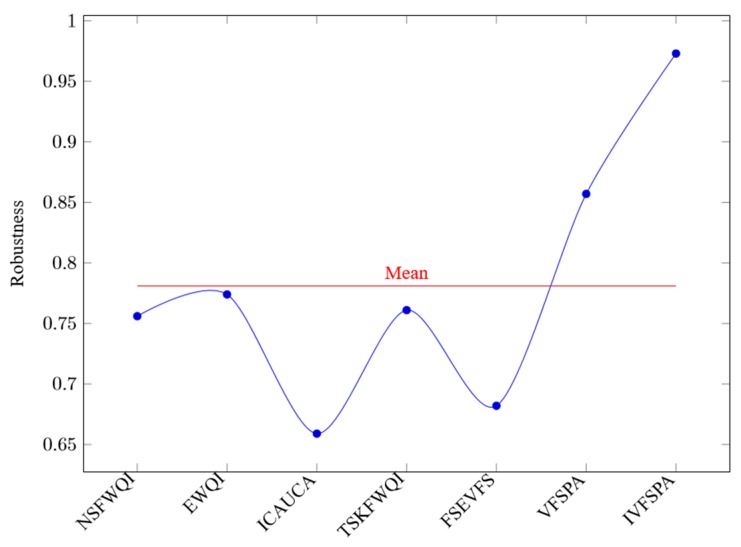
Robustness values.

In Figure 7, the horizontal axis represents the various methods, and the vertical axis represents the corresponding robustness values. Here, the robustness mean of all methods is 0.781, and the robustness values of NSFWQI, EWQI, ICAUCA, TSKFWQI, FSEVFS, VFSPA and IVFSPA are 0.756, 0.774, 0.659, 0.761, 0.682, 0.857 and 0.973, respectively. Obviously, of all experiments, the proposed method IVFSPA outperforms other assessment methods.

**Figure 8:**
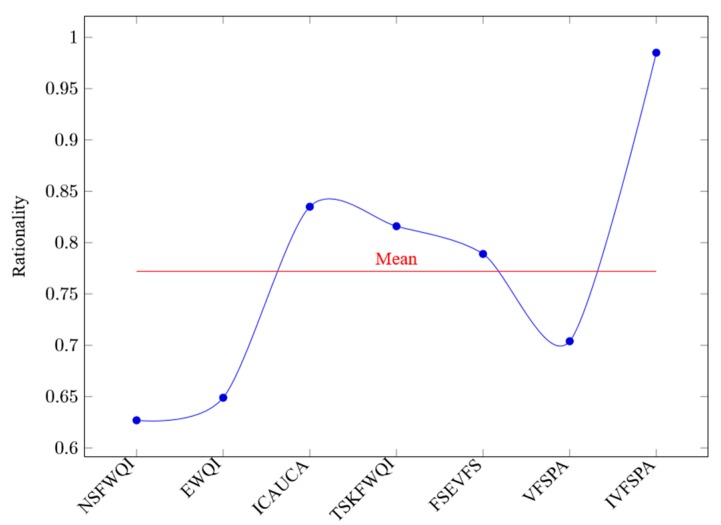
Rationality values.

In Figure 8, the horizontal axis represents the various methods, and the vertical axis represents the values of rationality. Of all experiments, our IVFSPA method has the best performance of rationality, which achieves the highest rationality value (0.985) of all methods. Meanwhile, the rationality values of NSFWQI, EWQI, ICAUCA, TSKFWQI, FSEVFS and VFSPA are 0.627, 0.649, 0.835, 0.816, 0.789 and 0.704, respectively. Thus, the rationality mean of all methods is 0.772.

**Figure 9:**
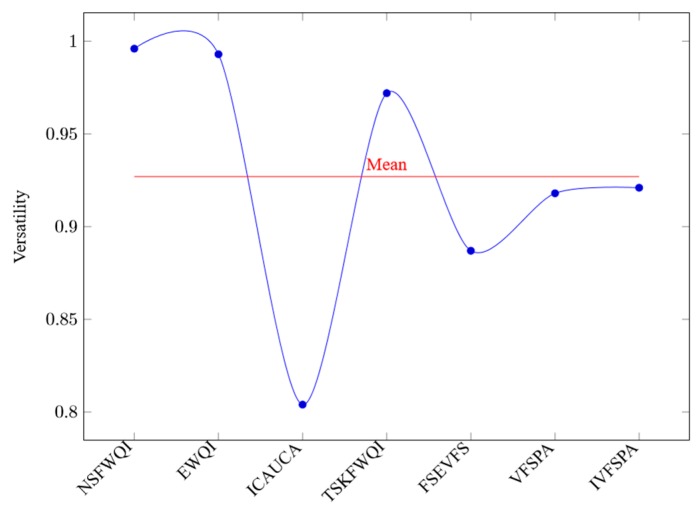
Versatility values.

In Figure 9, the horizontal axis represents the various methods, and the vertical axis represents the corresponding versatility values. It is easy to find that the versatility values of NSFWQI, EWQI, ICAUCA, TSKFWQI, FSEVFS, VFSPA and IVFSPA are 0.996, 0.993, 0.804, 0.972, 0.887, 0.918 and 0.921, respectively. Therefore, the versatility mean of all methods is 0.927. Obviously, in the most experiments, compared with other six evaluation methods, we can see that our IVFSPA obtains medium level of versatility for water quality assessment. The reason for being inferior to the methods of NSFWQI, EWQI and TSKFWQI may be that IVFSPA has a complex and holistic evaluation model.

Furthermore, in order to make the comparison more clearly, we consider the data analysis according to the following two tables (Table 6 and Table 7). All experimental results of metrics validation are shown in Table 6. It is clear that, in terms of precision (0.995), robustness (0.973) and rationality (0.985), the proposed IVFSPA has the best performance. In addition, in terms of the metrics of versatility (0.921), the proposed IVFSPA also has a good ability. They are the means of precision, which are the other evidences of performance. The precision mean of IVFSPA is 0.995, which is the highest precision mean of all. The precision means of NSFWQI, EWQI, ICAUCA, TSKFWQI, FSEVFS and VFSPA are 0.819, 0.832, 0.889, 0.950, 0.798 and 0.867, respectively.

Experimental detailed results of precision are shown in Table 7. From Table 7, we can easily see that the precision values of IVFSPA of representative sampling locations are 0.997, 1, 0.995, 0.998, 0,989, 0.991, 0.993, 1, 1, 0.997, 0.994, 0.988, 0.992 and 0.995, respectively. Obviously, those are the highest precision values of all assessment methods. Especially, on the sampling locations of “Ch-lh section/Chuhe gate” and “Lishui River/Kaitai bridge”, IVFSPA has a far higher level of precision compared with the other eavluation methods. All experimental results of metrics validation show that our proposed IVFSPA has the best performance.

## 5. Conclusions

In this paper, we propose an improved variable fuzzy set pair analysis (IVFSPA) method in light of the theory of set pair analysis and variable fuzzy set and design an optimized fusion architecture for assessment of contaminated surface waters. The proposed IVFSPA method can take into account the reasonable weight in each variable factor for every surface water assessment index and can clearly optimize traditional assessment methods used to date since the traditional assessment methods often employ single weights, not comprehensive weights. Then, we develop an improved Nemerow index method, which has improved the arithmetic form of $P_{i},$ to make the IVFSPA method more prominent and balanced. In the assessment process, a double judgment mode is employed in view of the principle of maximum membership degree and the positive and negative relationship which is built on the way of the monitored value and the standard value. Thus, this proposed IVFSPA method can improve the precision, robustness, rationality and versatility of assessments. To verify the performance of the proposed method, experiments are implemented on data from 14 major river collection sections at Nanking in 2017 and the results are compared with the other six assessment methods. The results indicate that our IVFSPA method can enhance the precision, robustness, rationality and versatility and is superior for the evaluation of polluted surface waters. Therefore, the IVFSPA method proposed in this study is more suitable as a comprehensive assessment tool for surface water quality in complex hydrological environment, especially for drinking water quality assessment.

## Figures and Tables

**Table 1:** Characteristics of fuzzy synthesis operator.

	Expression	Fuzzy Synthesis Operator			
Characteristic		*M* (●,+)	*M* (●,∨)	*M* (∧,⊕)	*M* (∨,∧)
Feature efficiency		Weighted Average Significant	Highlight main Cause	Highlight main Main cause	Highlight main Main cause
Weight effect			Significant	Not Significant	Not Significant
R function		Sufficient use	Insufficient	Under sufficient	Insufficient
Overall effect		Very strong	Weak	Very strong	Weak

**Table 2:** The measured data of surface waters in Nanking during the 2017 flood.

River Section/Collection Point	TP	NH_3_-N	Pb	As	KMnO_4_	FC (K/L)	DO	COD	BOD_5_
Qinhuai River/Qiqiaowen	0.417	3.56	0.13	0.82	6.52	35	3.53	22.87	4.26
Guanxi River/Qianjiadu	0.386	3.14	0.11	0.57	6.71	22	3.21	22.12	4.37
Chuhe jp section/Chenqian	0.412	2.68	0.08	0.34	7.04	32	3.13	20.24	5.29
Chuhe lh section/Chuhe gate	0.415	3.77	0.07	0.23	7.12	41	3.1	20.56	5.03
Yuhuai section/Jiezhi gate	0.405	3.32	0.05	0.04	6.65	39	3.09	22.33	6.01
Jiangning section/Yang bridge	0.433	2.39	0.09	0.15	6.44	27	3.51	21.13	5.88
Lishui section/Wusha bridge	0.419	3.66	0.07	0.56	7.37	34	3.22	23.27	5.42
Jurong River/Tu bridge	0.403	3.45	0.08	0.33	6.77	31	3.24	24.88	5.79
Chang jiang/Nanking bridge	0.376	3.39	0.08	0.4	6.82	36	3.27	21.36	5.5
Lishui River/Kaitai bridge	0.421	3.8	0.06	0.46	7.02	42	3.06	20.41	5.28
Shize River/Tian bridge	0.406	3.58	0.07	0.44	6.73	35	3.17	20.93	5.62
Chang jiang/	0.301	2.55	0.05	0.16	6.23	21	3.3	21.01	5.07
Jiangning estuary									
Chang jiang/	0.304	2.73	0.06	0.15	6.49	23	3.42	21.15	5.26
Jiuxiang estuary									
Meishan section/shore zone	0.422	3.39	0.08	0.67	7.52	24	3.26	23.94	5.91

Note that, the units are mg/L (the same below). The geology and geographic location of the research area are shown in Figure 3.

**Table 3:** Surface water quality standard.

Serial number	Index/Element	Class I	Class II	Class III	Class IV	Class V
1	TP	≤0.02	≤0.1	≤0.2	≤0.3	≤0.4
2	NH3-N	≤0.15	≤0.5	≤1	≤1.5	≤2
3	Pb	≤0.01	≤0.01	≤0.05	≤0.05	≤0.1
4	As	≤0.01	≤0.05	≤0.05	≤0.1	≤0.1
5	KMnO4	≤2	≤4	≤6	≤10	≤15
6	FC(K/L)	≤0.2	≤2	≤10	≤20	≤40
7	DO	≥7.5	≥6	≥5	≥3	≥2
8	COD	≤15	≤15	≤20	≤30	≤40
9	BOD5	≤3	≤3	≤4	≤6	≤10

**Table 4:** Result of the IVFSPA approach.

River Section/Collection Point	Class I	Class II	Class III	Class IV	Class V	Evaluation Level
Qinhuai River/Qiqiaowen	0.1921	0.1879	0.1899	0.2713	0.2066	V
Guanxi River/Qianjiadu	0.1641	0.1757	0.1908	0.2705	0.1989	V
Chuhe jp section/Chenqian	0.143	0.1981	0.201	0.2633	0.1946	IV
Ch lh section/Chuhe gate	0.144	0.2001	0.1783	0.2719	0.2057	V
Yuhuai section/Jiezhi gate	0.2167	0.1422	0.1942	0.2537	0.1932	IV
Jiangn section/Yang bridge	0.1785	0.1661	0.1955	0.2612	0.1987	IV
Lish section/Wusha bridge	0.0972	0.2135	0.2004	0.2236	0.2653	V
Jurong River/Tu bridge	0.15	0.1895	0.1787	0.2705	0.2113	V
Chang jiang/Nanking bridge	0.1706	0.157	0.1945	0.2534	0.2245	V
Lishui River/Kaitai bridge	0.1219	0.1853	0.1985	0.2406	0.2537	V
Shize River/Tian bridge	0.1225	0.1624	0.2007	0.2728	0.2416	V
Chang jiang/Jiangn estuary	0.1183	0.1974	0.2391	0.2526	0.1927	IV
Chang jiang/Jiuxiang estuary	0.1426	0.2011	0.1976	0.2604	0.1983	IV
Meishan section/shore zone	0.1125	0.1648	0.2107	0.2355	0.2765	V

**Table 5:** Operation results of different approaches.

Approach	NSFWQI	EWQI	ICAUCA	TSKFWQI	FSEVFS	VFSPA	IVFSPA
Qinhuai River/Qiqiaowen	V	IV	IV	V	V	V	V
Guanxi River/Qianjiadu	IV	V	V	V	V	IV	V
Chuhe jp section/Chenqian	III	IV	IV	III	IV	IV	IV
Ch lh section/Chuhe gate	IV	IV	IV	IV	IV	IV	V
Yuhuai section/Jiezhi gate	IV	IV	V	IV	IV	IV	IV
Jiangn section/Yang bridge	IV	IV	IV	V	IV	IV	IV
Lish section/Wusha bridge	V	V	V	V	V	V	V
Jurong River/Tu bridge	V	V	V	V	IV	V	V
Chang jiang/Nanking bridge	V	V	V	V	V	V	V
Lishui River/Kaitai bridge	IV	IV	IV	IV	IV	IV	V
Shize River/Tian bridge	V	V	V	V	V	V	V
Chang jiang/Jiangn estuary	IV	III	IV	IV	IV	IV	IV
Chang jiang/Jiuxiang estuary	IV	IV	III	IV	IV	IV	IV
Meishan section/shore zone	V	V	V	V	IV	V	V

**Table 6:** Experimental results of metrics validation.

Method	Precision	Robustness	Rationality	Versatility
NSFWQI	0.819	0.756	0.627	0.996
EWQI	0.832	0.774	0.649	0.993
ICAUCA	0.889	0.659	0.835	0.804
TSKFWQI	0.95	0.761	0.816	0.972
FSEVFS	0.798	0.682	0.789	0.887
VFSPA	0.867	0.857	0.704	0.918
IVFSPA	0.995	0.973	0.985	0.921

**Table:** 

(a)							
Approach	Qinhuai River/Qiwenqiao	Guanxi River/Qianjiadu	Chuhejp Section/Chenqian	Ch lh Section/Chuhe Gate	Yuhuai Section/Jiezhi Gate	Jiangn Section/Yang Bridge	Lish Section/Wusha Bridge
NSFWQI	0.884	0.821	0.816	0.683	0.825	0.882	0.819
EWQI	0.897	0.885	0.897	0.532	0.873	0.894	0.863
ICAUCA	0.933	0.937	0.927	0.673	0.918	0.928	0.923
TSKFWQI	0.995	0.992	0.918	0.865	0.937	0.967	0.978
FSEVFS	0.821	0.812	0.816	0.655	0.822	0.808	0.833
VFSPA	0.895	0.891	0.884	0.817	0.846	0.876	0.887
IVFSPA	0.997	1	0.995	0.998	0,989	0.991	0.993

**Table:** 

(b)							
Approach	Jurong River/Tu Bridge	Chang Jiang/Nanking Bridge	Lishui River/Kaitai Bridge	Shize River/Tian Bridge	Chang jiang/Jiangn Estuary	Chang jiang/Jiuxiang Estuary	Meishan Section/Shore Zone
NSFWQI	0.853	0.848	0.706	0.827	0.835	0.807	0.856
EWQI	0.867	0.883	0.517	0.902	0.879	0.885	0.877
ICAUCA	0.931	0.93	0.639	0.935	0.912	0.924	0.939
TSKFWQI	0.982	0.977	0.825	0.948	0.956	0.972	0.989
FSEVFS	0.813	0.836	0.634	0.826	0.827	0.838	0.834
VFSPA	0.879	0.893	0.804	0.829	0.867	0.901	0.862
IVFSPA	1	1	0.997	0.994	0.988	0.992	0.995
